# In Vitro Antifungal and Wound-Healing Potential of *Ferulago cassia* and *Ferulago silaifolia* Essential Oils in Skin Candidiasis

**DOI:** 10.3390/antibiotics15050471

**Published:** 2026-05-06

**Authors:** Carolina Furtado, Manuel González-Vázquez, Ceyda Sibel Kılıç, Lígia Salgueiro, Mónica Zuzarte

**Affiliations:** 1 Faculty of Pharmacy, University of Coimbra, 3000-548 Coimbra, Portugal; carolinafurtado99@gmail.com (C.F.); ligia@ff.uc.pt (L.S.); 2 Department of Pharmacology, Faculty of Pharmacy, University of Seville, 41012 Seville, Spain; mgonzalez15@us.es; 3 Department of Pharmaceutical Botany, Faculty of Pharmacy, Ankara University, 06560 Ankara, Türkiye; erdurak@pharmacy.ankara.edu.tr; 4 Chemical Engineering and Renewable Resources for Sustainability (CERES), Department of Chemical Engineering, Faculty of Sciences and Technology, University of Coimbra, 3030-790 Coimbra, Portugal; 5 Coimbra Institute for Clinical and Biomedical Research (iCBR), Faculty of Medicine, University of Coimbra, 3000-548 Coimbra, Portugal; 6 Center for Innovative Biomedicine and Biotechnology (CIBB), University of Coimbra, 3000-548 Coimbra, Portugal; 7 Clinical Academic Centre of Coimbra (CACC), 3000-076 Coimbra, Portugal

**Keywords:** *Candida albicans*, germ tube, biofilm, fibroblasts, tyrosinase

## Abstract

**Background/Objectives:** Skin candidiasis is a key contributor to chronic, non-healing wounds, largely due to persistent microbial infections. *Candida* species can colonize the skin, form protective biofilms, and interfere with enzyme activity, leading to extracellular matrix degradation, changes in pigmentation, and impaired wound healing. The rising prevalence of antifungal resistance challenges its management, underscoring the need for more effective antifungal therapies. Therefore, this study aimed to assess the antifungal effects and wound-healing potential of essential oils (EOs) from *Ferulago* spp. **Methods:** The antifungal activity of the EOs from five *Ferulago* species was evaluated against *Candida* spp. and *Cryptococcus neoformans*. The most active EOs were further investigated for their effects on *C. albicans* virulence factors, including germ tube formation, as well as biofilm formation and disruption. These effects were assessed using microscopic observation, XTT reduction assay, and crystal violet and safranin stainings. The wound-healing potential of the EOs was evaluated using the scratch-wound assay on fibroblasts and keratinocytes. Additionally, the effect on tyrosinase and elastase activity, was also investigated. **Results:** *F. silaifolia* and *F. cassia* essential oils showed fungicidal activity against *Candida* spp. and *Cryptococcus neoformans*. *F. silaifolia* displayed greater potency, with lower MIC and MLC values. Both oils inhibited key *C. albicans* virulence factors at sub-MIC concentrations. *F. silaifolia* EO was more effective in preventing biofilm formation whereas *F. cassia* EO showed notable tyrosinase inhibitory effect. **Conclusions:** These findings align with traditional uses and suggest that *F. silaifolia* and *F. cassia* EOs exhibit antifungal activity alongside properties associated with wound healing, supporting their potential as topical antifungal agents and thereby justifying further investigation.

## 1. Introduction

The skin is a multifunctional organ that serves as a vital barrier against microorganisms, UV radiation, and mechanical injury, while also providing sensory perception [1,2], regulating body temperature and contributing to immune defense [3]. Despite these essential functions, skin injury triggers a complex healing cascade that can become impaired, leading to wound formation. Such wounds represent a serious yet frequently overlooked complication of many conditions, ranging from diaper dermatitis in infants and intertrigo in skin folds to chronic wounds in patients with diabetes or burn injuries [4]. One of the major factors that contributes to compromised wound healing is the formation of microbial biofilms within the wound environment. These structured communities of microorganisms disrupt the highly organized and complex process of tissue regeneration, ultimately resulting in chronic wounds [5]. A chronic wound remains trapped in the inflammatory phase and fails to achieve complete tissue and functional recovery [6]. A key contributor to this persistent inflammatory state is biofilm-associated infection. This risk is particularly high in immunocompromised patients, whose weakened immune defenses facilitate fungal invasion of tissues and lead to persistent, difficult-to-treat infections [7]. Although research on wound infections has largely focused on bacteria, growing evidence highlights the contribution of fungi and emphasizes the emerging importance of the wound microbiome [8]. Moreover, fungal involvement in non-healing ulcers facilitates bacterial persistence by enhancing resistance to both antibiotics and host immune responses, thereby transforming the wound into a polymicrobial environment, resistant to treatment [9].

Among fungal pathogens, *Candida albicans* plays a prominent role in chronic wound infections. *C. albicans* is an opportunistic fungus that normally exists as a commensal organism on the skin and mucous membranes. However, under conditions of immune imbalance, it can become pathogenic, leading to systemic invasive candidiasis that may affect the bloodstream, bones, and even the brain [9,10]. Notably, *C. albicans* is included in the World Health Organization list of priority fungal pathogens due to its significant global health burden and increasing antifungal resistance [11]. Other clinically important *Candida* species, such as *C. glabrata*, *C. tropicalis*, *C. parapsilosis*, and the emerging multidrug-resistant *C. auris*, also contribute to invasive infections and biofilm-associated antifungal resistance, further complicating wound healing and clinical management [12,13]. These infections can also interfere with key physiological mechanisms involved in tissue repair. In healthy wounds, matrix metalloproteinases play an important role, degrading damaged extracellular matrix components and facilitating proper re-epithelialization. In contrast, chronic wounds often exhibit excessive protease activity, including elevated elastase levels, which degrade healthy tissue and maintain the wound in a persistent inflammatory state [14]. In addition, other skin enzymes also contribute to cutaneous physiological processes, such as tyrosinase, a copper containing oxidase enzyme involved in the production of melanin. Recent investigations revealed that tyrosinase inhibitors are potential antimicrobial agents, due to their ability to disrupt cell membrane integrity, interfere with metabolic enzymes, and sequester essential metal ions required for bacteria [15].

In light of the rising prevalence of fungal infections, particularly those caused by *Candida* species, and the growing concern of antifungal resistance, the development of novel therapeutic strategies has become increasingly important.

In this context, aromatic plants have emerged as promising sources of bioactive compounds, namely essential oils, offering potential alternative or adjuvant antifungal therapies [16]. Additionally, some essential oils exhibit enzyme inhibitory activity, which may provide further therapeutic benefits in wound care and ultimately enhance patients’ quality of life. Building on previous findings demonstrating that essential oils from several *Ferulago* species have antifungal potential [17,18,19,20,21,22,23,24,25], the present study aimed to investigate its effect against yeasts.

*Ferulago* W.D.J.M. Koch is an important genus within the Apiaceae family, comprising approximately 48 taxa worldwide [26]. Species of this genus are mainly distributed in Türkiye, Iraq, and western Iran. Of the approximately 35 taxa recorded in Türkiye, 19 are endemic, making the country a recognized centre of genetic diversity for this genus [27,28,29]. Regarding traditional use, species of the genus *Ferulago* have long been employed in the treatment of skin wounds and infections. In addition, these plants have been used as natural food preservatives to extend shelf life, suggesting relevant antimicrobial properties [29]. Traditional uses of *Ferulago* species are well documented, particularly in Iran and Türkiye, where various plant parts, including aerial parts and essential oils, are commonly used for dermal applications. For example, *F. angulata* is traditionally used in Iran for the treatment of dermal wounds, being regarded as a wound healer and antiseptic [30,31], while *F. carduchorum* is applied as a poultice for similar purposes in southeastern Iran [32]. The essential oil of *F. angulata* fruits is also incorporated into topical anti-inflammatory and analgesic ointments, balms, and creams, reflecting its bioactive potential [33], whereas *F. trachycarpa* from the Tokat region has been traditionally used as an antiseptic [34].

In the present study, a screening assay was performed on five *Ferulago* species from Türkiye: *F. cassia*, *F. isaurica*, *F. setifolia*, *F. silaifolia*, and *F. syriaca*. Among them, the endemic species *F. silaifolia* and *F. cassia* exhibited the highest antifungal activity and, therefore, were selected for further evaluation of their effects on *Candida albicans* virulence factors, including germ tube formation and biofilm development, as well as their potential impact on fibroblast and keratinocyte migration and key skin-related enzyme activity.

## 2. Results

### 2.1. Chemical Composition

Gas chromatography and gas chromatography–mass spectrometry analyses revealed that the major volatile constituents of *F. cassia* were α-pinene (61.0%) and cis-chrysanthenyl acetate (21.3%) whereas those of *F. silaifolia* were α-pinene (45.4%) and cis-chrysanthenyl acetate (39.1%). The detailed composition of these essential oils and the others used in this work has been described previously [35].

### 2.2. Antifungal Potential of Ferulago spp. Essential Oils

#### 2.2.1. Minimal Inhibitory and Minimal Lethal Concentrations

A brief screening assay was carried out to assess the antifungal potential of the essential oils (*F. cassia, F. isaurica*, *F. setifolia*, *F. silaifolia* and *F. syriaca*) against *Candida* spp. and *Cryptococcus neoformans*. The most active species, presenting lower minimal inhibitory concentrations (MIC) and minimal lethal concentrations (MLCs) were *F. cassia* and *F. silaifolia*. Overall, *F. silaifolia* was the most effective essential oil showing fungicidal effects against all the tested strains, with particular strong activity against *C. neoformans* (MIC = 50 μg/mL), *C. krusei*, *C guilliermondii* and *C parapsilosis* (MIC = 100 μg/mL). *F. cassia* was also very effective against these strains, although presenting slightly higher MIC and MLC values. Table 1 summarizes the antifungal activity of both essential oils against different strains of *Candida* spp. and *C. neoformans*, expressed as MIC, MLC, MIC_50_, and MIC_90_ values. In addition, a sorbitol assay was performed for *C. albicans*; however, no alteration in MIC values was observed in the presence of sorbitol suggesting that the antifungal activity of both essential oils is unlikely to be associated with direct disruption of the fungal cell wall integrity.

**Figure 1:**
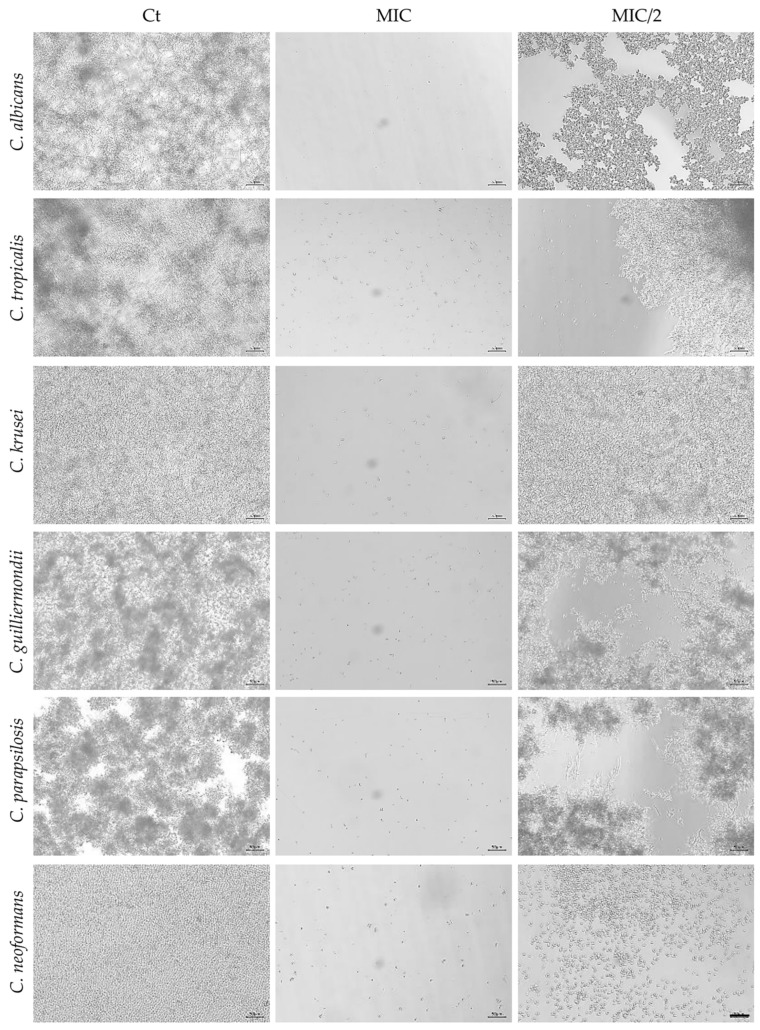
Representative microscopic images demonstrating growth inhibition in the tested fungal species following exposure to *F. silaifolia* essential oil. Ct—control; MIC—Minimal Inhibitory Concentration as shown in Table 1; MIC/2—half the MIC; scale bar = 50 μm.

**Figure 2:**
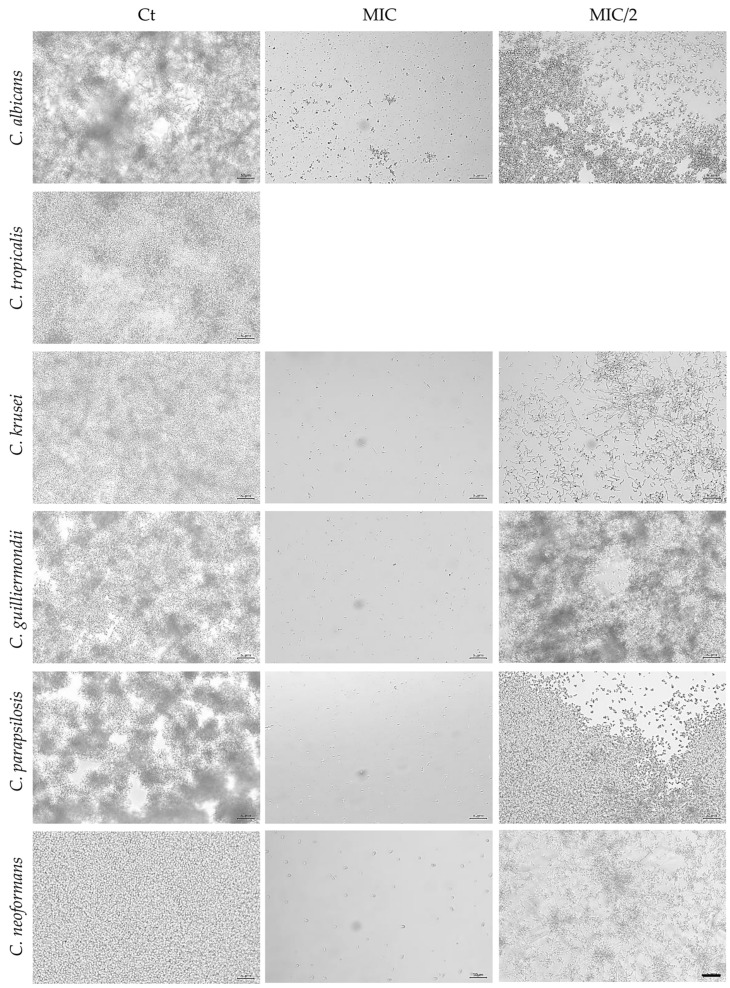
Representative microscopy images demonstrating growth inhibition in the tested fungal species following exposure to *F. cassia* essential oil. Ct—control; MIC—Minimal Inhibitory Concentration as shown in Table 1; MIC/2—half the MIC; scale bar = 50 μm.

Optical microscopy images were also obtained and were consistent with the results described above. As expected, no fungal growth was observed at MIC concentrations compared with the control, whereas partial fungal growth was detected at half the MIC concentration (MIC/2; Figure 1 and Figure 2). When MIC values exceeded 800 µg/mL (the highest essential oil concentration used), only a representative image of the positive control is shown (Figure 2).

#### 2.2.2. Effect on Germ Tube Formation in *Candida albicans*

**Figure 3:**
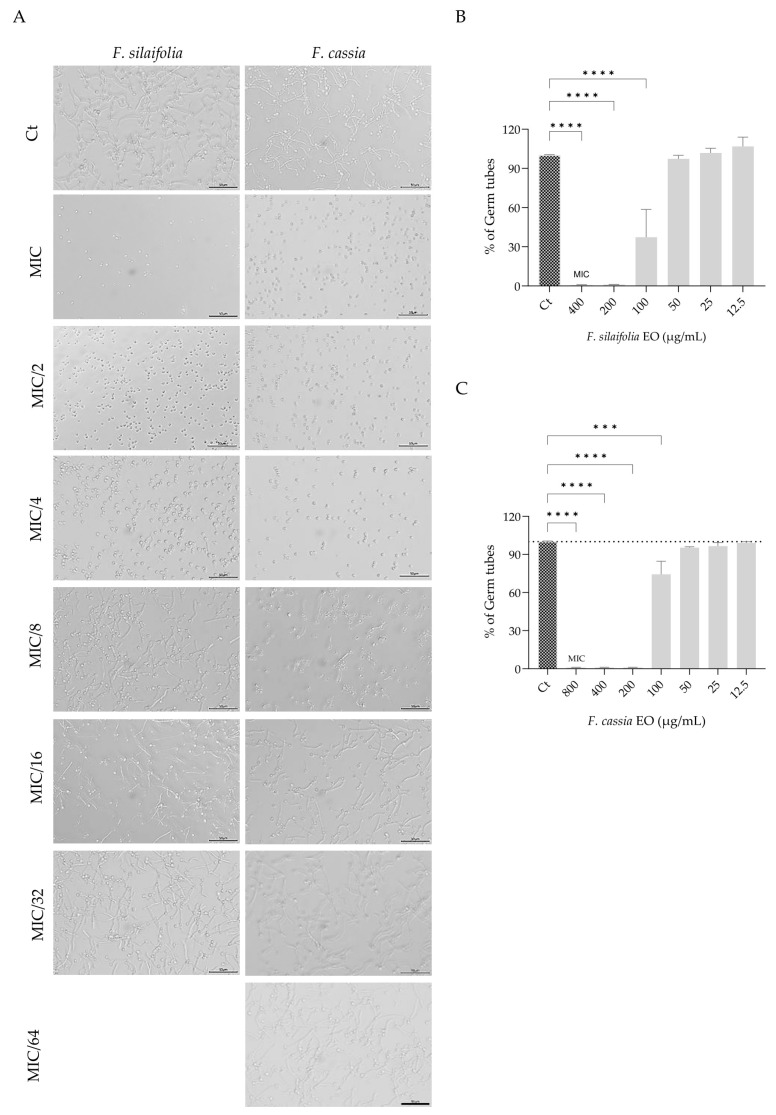
Yeast to hypha transition in *C. albicans* in the presence of MIC and sub-inhibitory concentrations of *F. silaifolia* and *F. cassia* essential oils. (**A**) Representative microscopy images. (**B**,**C**) Quantification of the percentage of germ tubes formed following treatment with *F. silaifolia* (**B**) and *F. cassia* (**C**) essential oils. Results expressed as mean ± SEM of at least three independent experiments. Statistical analysis was performed using one-way ANOVA followed by Dunnett’s multiple comparisons test. Statistical significance is indicated as *** *p* < 0.001 and **** *p* < 0.0001, compared to the control group. Scale bar = 50 μm.

Since germ tube formation is one of the most relevant virulence factors in *Candida albicans,* the effect of *F. silaifolia* and *F. cassia* essential oils on the yeast to hypha transition was assessed. Figure 3A shows representative microscopic images of fungal control cultures and cultures exposed to the essential oil at the MIC and sub-inhibitory concentrations (MIC/2–MIC/64). It is quite evident that both essential oils were able to inhibit the germ tube formation in concentrations well below their MIC (400 µg/mL for *F. silaifolia* and 800 µg/mL for *F. cassia*), showing a complete inhibition of germ tube formation at 200 µg/mL, Figure 3B,C). Interestingly, *F. cassia*, although presenting a higher MIC, was very effective in decreasing germ tube formation, a virulence associated trait, and remained significantly effective even at MIC/8 (100 µg/mL; Figure 3C).

#### 2.2.3. Effect on *Candida albicans* Early-Stage Biofilm Formation

**Figure 4:**
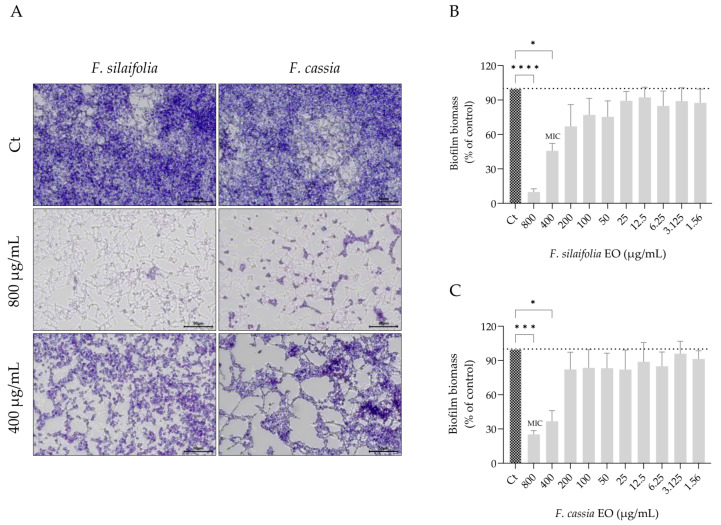
Immature biofilms, untreated (control—Ct) or treated with essential oils from *F. silaifolia* and *F. cassia*. (**A**) Representative microscopy images of biofilm biomass stained with crystal violet. (**B**,**C**) Quantification of biofilm biomass following treatment with *F. silaifolia* (**B**) and *F. cassia* (**C**) essential oils. Results expressed as mean ± SEM of at least three independent experiments. Statistical analysis was performed using one-way ANOVA followed by Dunnett’s multiple comparisons test. Statistical significance is indicated as * *p* < 0.05, *** *p* < 0.001 and **** *p* < 0.0001, compared to control group. Scale bar = 50 μm.

**Figure 5:**
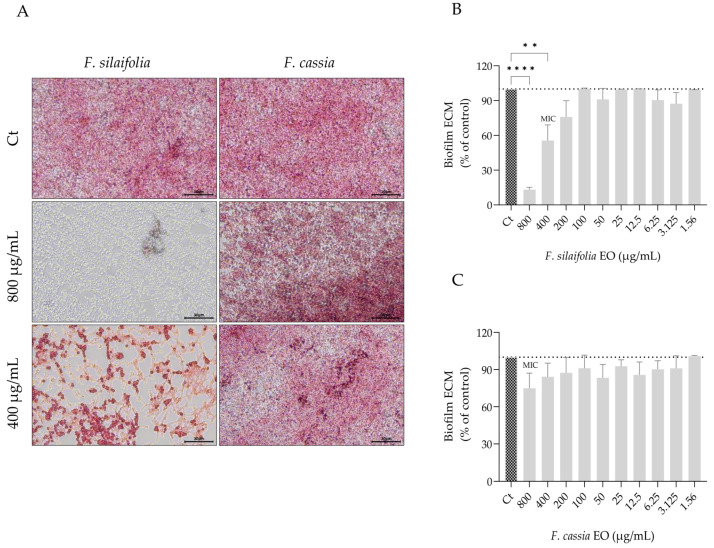
Immature biofilms, untreated (control—Ct) or treated with essential oils from *F. silaifolia* and *F. cassia*. (**A**) Representative microscopy images of biofilm extracellular matrix stained with safranin red. (**B**,**C**) Quantification of biofilm matrix following treatment with *F. silaifolia* (**B**) and *F. cassia* (**C**) essential oils. Results expressed as mean ± SEM of at least three independent experiments. Statistical analysis was performed using one-way ANOVA followed by Dunnett’s multiple comparisons test. Statistical significance is indicated as ** *p* < 0.01 and **** *p* < 0.0001, compared to the control group. Scale bar = 50 μm.

Similar to germ tube formation, biofilm formation also contributes to *C. albicans* virulence and antifungal resistance. For this reason, the effect of both essential oils on three distinct biofilm parameters was assessed, namely biofilm biomass, extracellular matrix deposition and fungal viability within the biofilm. The essential oils altered biofilm architecture and morphology, as observed in representative microscopy images (Figure 4A and Figure 5A), indicating changes in biofilm organization and structural connectivity.

**Figure 6:**
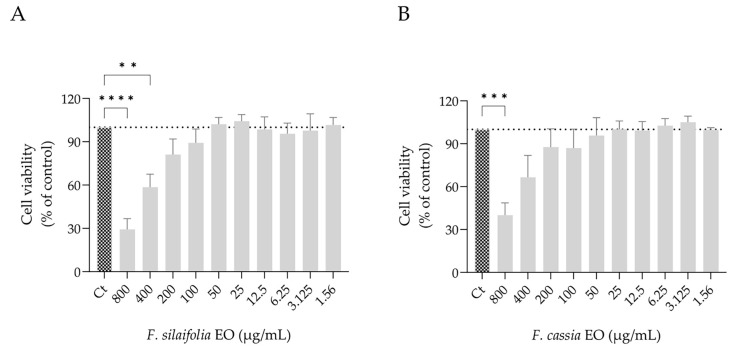
Immature biofilms, untreated (control—Ct) or treated with essential oils from *F. silaifolia* and *F. cassia*. (**A**,**B**) Quantification of biofilm viability by XTT assay following treatment with *F. silaifolia* (**A**) and *F. cassia* (**B**) essential oils. Results expressed as mean ± SEM of at least three independent experiments. Statistical analysis was performed using one-way ANOVA followed by Dunnett’s multiple comparisons test. Statistical significance is indicated as ** *p* < 0.01, *** *p* < 0.001 and **** *p* < 0.0001, compared to the control group.

Quantitative assays further demonstrated that *F. silaifolia* essential oil was highly effective at 400 μg/mL, leading to a reduction of around 50% in both biofilm biomass (Figure 4B) and extracellular matrix deposition (Figure 5B). These findings highlight its potential as a natural strategy against biofilms, another important virulence factor of *C. albicans*. *F. cassia* essential oil was also effective, producing similar reductions in biofilm biomass (Figure 4C). However, in contrast to *F. silaifolia*, it was less effective at reducing extracellular matrix deposition, achieving only a 16% reduction at 400 μg/mL (Figure 5C). The effects of both essential oils on *C. albicans* viability were also quantified (Figure 6). Consistent with the extracellular matrix results, *F. silaifolia* essential oil was more effective, significantly reducing the metabolic activity of the fungal cells by 50% at 400 µg/mL (Figure 6A). In contrast, *F. cassia* exhibited a weaker effect, decreasing fungal viability by 30% (Figure 6B).

#### 2.2.4. Effect on *Candida albicans* Mature Biofilm Disruption

Identifying effective treatment strategies capable of disrupting established biofilms remains a significant challenge. Indeed, preventing biofilm formation is generally more effective, as it targets the early stages of microbial attachment, when fungal cells are still in the planktonic state and therefore more susceptible to antifungal agents. In contrast, mature biofilms are highly organized, structured communities surrounded by a self-produced extracellular matrix that provides physical protection and significantly increases resistance to antifungal treatments.

**Figure 7:**
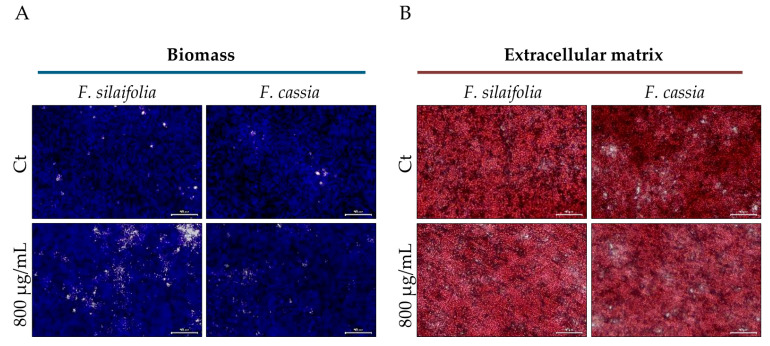
Mature biofilms, untreated (control) or treated with essential oils from *F. silaifolia* and *F. cassia*. (**A**) Representative microscopy images of biofilm biomass stained with crystal violet. (**B**) Representative microscopy images of biofilm extracellular matrix stained with safranin red. Scale bar = 50 μm.

In this study, we evaluated whether the tested essential oils had the ability to interfere with preformed biofilms. However, contrarily to that reported for biofilm formation, both essential oils were ineffective at this stage and did not disrupt mature biofilms, as shown in Figure 7A,B. Quantitative analyses were also conducted; however, as no differences were observed between the treated and control groups, these results are not presented.

### 2.3. Safety Profile on Fibroblasts and Keratinocytes

**Figure 8:**
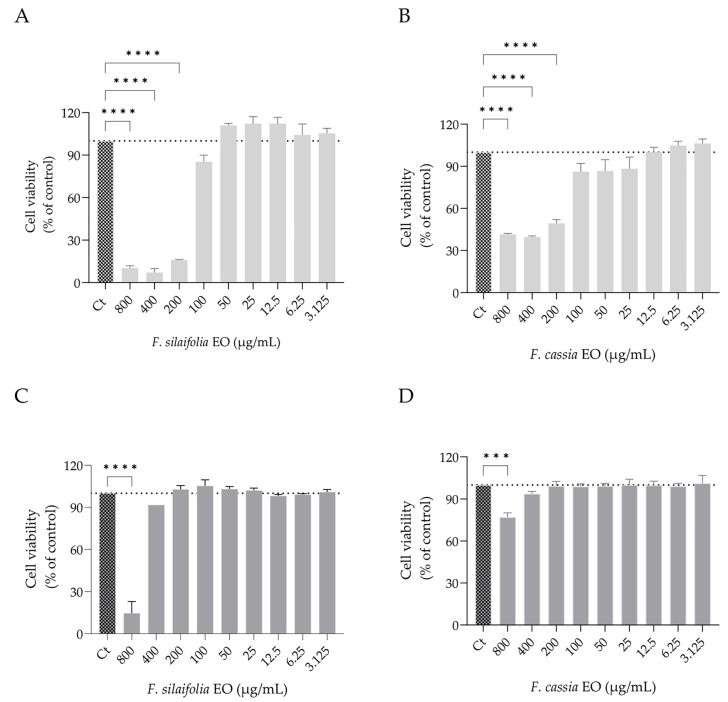
Effect of essential oils obtained from *F. silaifolia* and *F. cassia* on NIH 3T3 (**A**,**B**) and HaCaT (**C**,**D**) cell viability. Cells were treated with different concentrations of each essential oil and cell viability was assessed through resazurin reduction assay. Data are expressed as mean ± SEM of at least three independent experiments relative to untreated cells (control—100% viability). Statistical analysis was performed using one-way ANOVA followed by Dunnett’s multiple comparisons test. Statistical significance is indicated as *** *p* < 0.001, **** *p* < 0.0001, compared to control group (Ct).

The effect of the essential oils on both fibroblasts and keratinocytes viability was evaluated using the resazurin reduction assay (Figure 8). Keratinocytes, the primary cell type of the epidermis and initial cellular interface for topical application, showed no significant reduction in viability up to 400 µg/mL (Figure 8C,D). On the other hand, fibroblasts, located in the underlying dermis, exhibited higher sensitivity, with no detectable toxicity up to 100 µg/mL (Figure 8A,B), indicating a narrower safety margin for the essential oil in deeper skin layers. Accordingly, under the experimental conditions used, concentrations up to 400 µg/mL can be considered non-cytotoxic for keratinocytes, whereas concentrations up to 100 µg/mL were non-cytotoxic for fibroblasts.

### 2.4. Wound Healing Potential: Effect on Fibroblasts and Keratinocytes Migration

**Figure 9:**
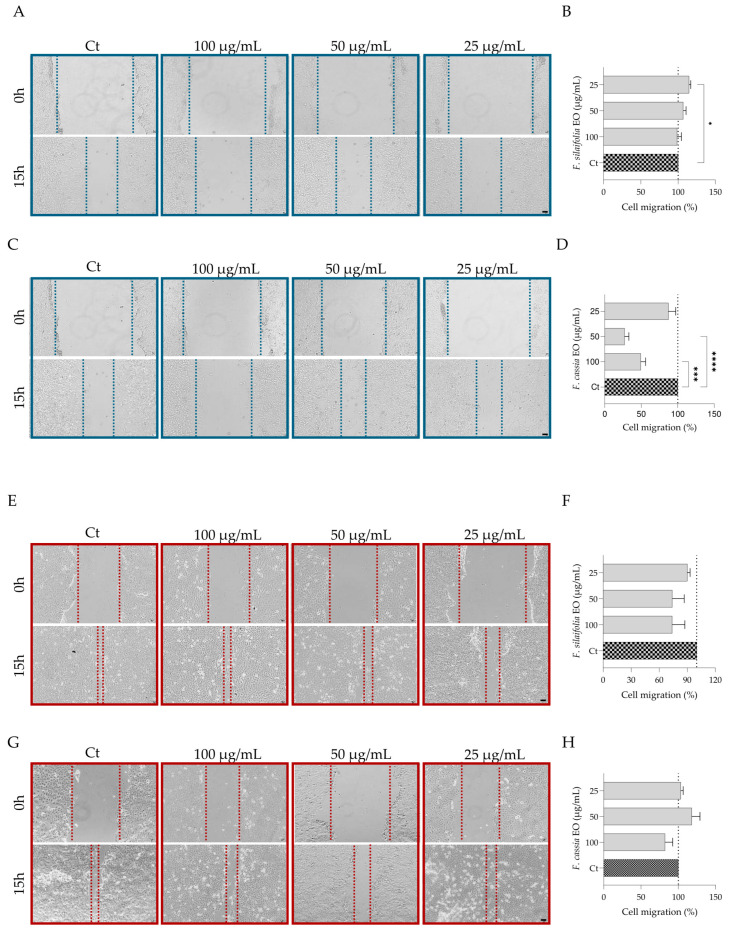
Effect of essential oils on NIH 3T3 (**A**–**D**) and HaCaT (**E**–**H**) migration. Cells were treated with different concentrations of the essential oil and migration was measured after 15 h following scratch induction. Representative microscopy images obtained at 0 h and 15 h after scratch induction and treatment with *F. silaifolia* (**A**,**E**) and *F. cassia* (**C**,**G**). Quantification of cell migration through wound area closure after 15 h in the presence of *F. silaifolia* (**B**,**F**) and *F. cassia* (**D**,**H**) essential oils. Data are expressed as mean ± SEM of at least three independent experiments and are presented relative to untreated cells (Ct—100% migration). Statistical analysis was performed using one-way ANOVA followed by Dunnett’s multiple comparisons test. Statistical significance is indicated as * *p* < 0.05, *** *p* < 0.001, **** *p* < 0.0001, compared to control group. Scale bar = 10 μm.

The scratch wound assay was used to evaluate the effect of the essential oils on cell migration, providing insight into their potential role in promoting wound healing. Both fibroblasts (Figure 9A–D) and keratinocytes (Figure 9E–H) were used, as they represent key contributors to wound healing, covering both re-epithelialization and tissue remodeling processes. Interestingly, lower concentrations of the essential oils appeared to be more effective in promoting cell migration, with statistically significant effects observed at 25 μg/mL of *F. silaifolia* essential oil in fibroblast migration (Figure 9A,B). Although statistical significance was not reached for *F. cassia* essential oil, a trend toward increased cell migration was observed, with 50 μg/mL showing the greatest effect (Figure 9G,H).

### 2.5. Enzyme Activity Inhibition Effects

**Figure 10:**
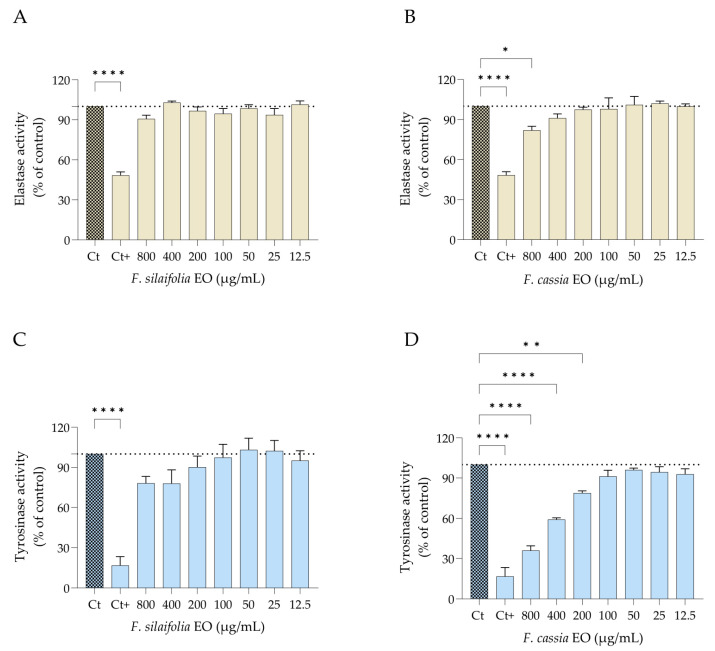
Effect of essential oils from *F. silaifolia* and *F. cassia* on skin enzyme activity. Elastase activity following treatment with *F. silaifolia* (**A**) and *F. cassia* (**B**). Tyrosinase activity following treatment with *F. silaifolia* (**C**) and *F. cassia* (**D**). Data represent mean ± SEM of at least three independent experiments and are expressed relative to the untreated control. Statistical analysis was performed using one-way ANOVA followed by Dunnett’s multiple comparisons test. Statistical significance is indicated as * *p* < 0.05, ** *p* < 0.01, **** *p* < 0.0001, compared to control group (Ct). Positive controls (Ct+) were quercetin (50 μg/mL) for elastase and kojic acid (10.6 mM) for tyrosinase.

Both elastase and tyrosinase activities were evaluated using an in chemico assay. Kojic acid and quercetin served as positive controls for tyrosinase and elastase assays, respectively, thereby confirming the validity of the experimental setup (Figure 10). *F. cassia* essential oil was the most effective, demonstrating significant inhibitory activity against both enzymes (Figure 10B,D). Notably, it showed strong inhibitory effect against tyrosinase, being effective even at low concentrations (200 μg/mL), with an IC_50_ of 543.23 ± 10.12 μg/mL (mean ± SD; Figure 10D). In contrast, the IC_50_ value was not determined for elastase inhibition, as the observed activity did not reach sufficient inhibition levels for curve fitting. Conversely, *F. silaifolia* essential oil showed no relevant inhibitory activity against either enzyme.

## 3. Discussion and Conclusions

Candidiasis refers to infections caused by *Candida* species, with *C. albicans* being the most clinically significant pathogen. This opportunistic yeast is generally harmless and part of the commensal flora of the skin and mucous membranes; however, under favorable conditions such as excess moisture, friction, or tissue damage, it can become pathogenic leading to localized or chronic infections [36]. In the skin and mucosa, candidiasis can manifest as intertrigo, diaper rash, oral thrush, paronychia, or chronic wound infections, with the ability to form germ tubes, hyphae, and biofilms that enable tissue invasion and antifungal resistance [37]. This opportunistic behavior makes candidiasis a significant clinical concern, particularly in immunocompromised patients, individuals with diabetes, or those with chronic wounds, where it can delay healing and adversely affect quality of life [38]. Another opportunistic fungus is *Cryptococcus neoformans*, primarily found in the environment, such as soil contaminated with bird droppings. *C. neoformans* usually does not colonize the skin, but can cause infections in immunocompromised patients, particularly those with HIV/AIDS, organ transplants, or prolonged corticosteroid use [39]. Both *C. albicans* and *Cryptococcus neoformans* are included in the World Health Organization (WHO) list of critical fungal pathogens due to their significant impact on human health [11]. Indeed, the growing prevalence of opportunistic fungal infections caused by these pathogens, combined with the rise in antifungal resistance highlights a critical gap in current treatment options. Therefore, investing in new therapeutic approaches based on effective natural compounds such as essential oils, can contribute to prevent treatment failure, and address this increasing global health burden.

Plants from the genus *Ferulago* have been traditionally used to treat infected wounds, snake bites and other dermatological conditions, revealing an antimicrobial and wound healing potential [29]. A recent study from our group explored the effects of *Ferulago* spp. essential oils in dermatophytosis. Among the tested species, *F. silaifolia* was able to inhibit fungal growth and biofilm formation especially in *Trichophyton rubrum* and promote fibroblast migration [25]. In light of these findings, we now intend to expand our study to candidiasis, focusing on yeast infections of the skin and mucous membranes.

In the present study, following an initial screening, both *F. silaifolia* and *F. cassia* essential oils emerged as the most effective, demonstrating fungicidal activity against both *Candida* species and *Cryptococcus neoformans*. *F. silaifolia* exhibited lower MIC and MLC values (50–400 vs. 100–800 μg/mL), indicating higher potency. Interestingly, both essential oils were able to inhibit *C. albicans* virulence factors, particularly germ tube formation, at concentrations well below their MIC values (100 μg/mL) but *F. silaifolia* was more effective in preventing biofilm formation, as it inhibited all measured parameters including biofilm biomass, extracellular matrix deposition, and fungal viability within the biofilm. In addition, a sorbitol protection assay performed on *C. albicans* showed no change in MIC values in the presence of sorbitol. Since sorbitol acts as an osmotic stabilizer that protects fungal cells with compromised cell walls, the absence of a MIC shift suggests that the antifungal activity of the essential oils is unlikely to involve direct disruption of fungal cell wall integrity. Regarding the disruption of mature biofilms, the essential oils did not show significant activity. This finding is consistent with the well-documented high tolerance of established biofilm structures to antimicrobial agents, which is largely attributed to their dense extracellular matrix and altered metabolic state. As a result, the eradication of preformed biofilms remains a major and persistent clinical challenge in the treatment of fungal infections.

In parallel, cytotoxicity assays showed that keratinocytes viability was not significantly affected up to 400 µg/mL, whereas fibroblasts displayed higher sensitivity, with essential oil-induced toxicity observed above 100 µg/mL. When considered alongside the antifungal MIC values, these results indicate that antifungal activity partially overlaps with the concentration range affecting fibroblasts, while remaining largely within the tolerance range of keratinocytes. This suggests a differential cellular response between epidermal and dermal cells upon essential oil exposure. However, direct comparison between MIC values and in vitro cytotoxicity should be interpreted with caution, as these endpoints reflect distinct biological systems and do not define a therapeutic window. Further in vivo studies are required to better establish safety and efficacy in a topical context.

To the best of the authors’ knowledge, this is the first report on the antifungal effects of *F. silaifolia* and *F. cassia* essential oils against *Candida* spp. and *Cryptococcus neoformans*. Previous studies have investigated essential oils from other *Ferulago* species against *C. albicans* using comparable methodologies. For example, *F. capillaris* essential oil was reported to inhibit *C. albicans* growth and germ tube formation at concentrations similar to those observed in the present study [17]. Likewise, essential oils from *F. asparagifolia*, *F. galbanifera*, *F. humilis*, and *F. trachycarpa* [40], as well as those from *Ferulago campestris* [41] have demonstrated comparable MIC values against *C. albicans*. However, these studies did not explore the underlying mechanisms of antifungal activity. Interestingly, although α-pinene is a major compound in all these essential oils, consistent with our findings for *F. silaifolia* and *F. cassia*, its activity against *C. albicans* does not fully account for the antifungal effects observed, suggesting that synergistic interactions among other constituents likely contribute to the enhanced antifungal effect [17,41].

Regarding cell migration, both fibroblasts and keratinocytes were considered in the present study as they represent complementary aspects of wound healing. Fibroblasts are critical for tissue repair, as their migration into the wound site enables extracellular matrix deposition and structural restoration. Keratinocytes, on the other hand, drive re-epithelialization by migrating across the wound surface to restore the skin barrier [42]. Notably, *F. silaifolia* essential oil at 25 μg/mL promoted fibroblast migration, supporting tissue repair and wound closure. This effect is particularly relevant in candidiasis, where *C. albicans* colonization and biofilm formation can damage tissue, prolong inflammation, and impair healing. By enhancing fibroblast migration, *F. silaifolia* essential oil not only contributes to control the infection but also accelerates the repair of damaged tissue, addressing a key secondary complication of candidiasis. As *F. silaifolia* was more effective in promoting fibroblast’s migration than keratinocytes migration, its primary action may be associated with enhancing tissue repair and extracellular matrix deposition rather than directly stimulating epithelial migration. A previous study carried out by our group also demonstrated the cell-migration potential of *F. lutea*, another species rich in α-pinene, although a higher concentration was required to induce fibroblasts migration (0.32 µL/mL vs. 25 µg/mL [43]).

Furthermore, the effect of the essential oils on the activity of key enzymes involved in the wound-healing process was evaluated providing additional insight into the potential mechanisms underlying the observed effects. In this context, *F. cassia* essential oil demonstrated strong tyrosinase inhibition, which is particularly relevant in wounds. By modulating tyrosinase-mediated oxidative reactions and consequent melanin production, this essential oil may help reduce inflammation, abnormal pigmentation, and oxidative stress, creating a more favorable environment for tissue repair, in addition to its antifungal activity.

Overall, these results support traditional knowledge and highlight the potential novelty of *F. silaifolia* and *F. cassia* essential oils as sources of bioactive compounds exhibiting both antifungal activity and wound-healing-related properties. These findings provide a valuable scientific basis for further research, contributing to the growing evidence on plant-derived antifungal agents and their potential relevance to candidiasis management and related applications. However, it is important to emphasize that the present findings are based on in vitro and in chemico assays and cannot be directly extrapolated to therapeutic efficacy, topical suitability, or wound-healing effects in vivo. Therefore, future studies, including in vivo models and clinical evaluation, are warranted to further validate their safety, efficacy, and translational potential.

## 4. Materials and Methods

### 4.1. Plant Material

Plant materials were collected from the localities summarized in Table 2 and taxonomically identified by Prof. Dr. Hayri Duman (Faculty of Science, Department of Biology, Gazi University). Voucher specimens are deposited in the Herbarium of Ankara University, Faculty of Pharmacy (AEF).

### 4.2. Essential Oils Isolation and Analysis

The essential oils were isolated by hydrodistillation from *Ferulago* spp. fruits and characterized by gas chromatography (GC) and gas chromatography–mass spectrometry (GC–MS), according to previously reported methodologies [35].

### 4.3. Antifungal Activity

#### 4.3.1. Fungal Strains

In order to evaluate the antifungal activity of essential oils from selected *Ferulago* species, both collection and clinical yeast strains were used, namely *Candida albicans* ATCC 10231, *Candida tropicalis* ATCC 13803, *Candida krusei* H9, *Candida guilliermondii* MAT23, *Candida parapsilosis* ATCC 90018 and *Cryptococcus neoformans* CECT 1078. Strains were cultured on Sabouraud dextrose agar (OXOID, Basingstoke, UK) and Potato dextrose agar (OXOID, Basingstoke, UK) plates at 35 °C.

#### 4.3.2. Fungal Growth

The impact of the essential oil on fungal growth was determined using two parameters: the minimal inhibitory concentration (MIC) and the minimal lethal concentration (MLC). These parameters were determined according to the EUCAST microdilution reference method for yeasts [44]. Briefly, the inoculum was prepared by adjusting the microbial suspension in sterile saline to 0.5 McFarland using a densitometer, followed by a 1:10 dilution in sterile water to obtain a final concentration of 1–5 × 10^5^ CFU/mL. A volume of 100 μL of the inoculum was added to each well, resulting in a final density of 0.5–2.5 × 10^5^ CFU/mL. Two-fold serial dilutions of the essential oils were prepared from a stock solution in DMSO and added to each well, yielding final concentrations ranging from 800 to 1.56 μg/mL (800, 400, 200, 100, 50, 25, 12.5, 6.25, 3.125 and 1.56 μg/mL). For each strain, a positive control (culture medium containing 100 μL of inoculum) and a negative control (culture medium without fungal inoculum) were included. The non-inhibitory effect of DMSO was confirmed at the highest concentration used in the essential oil dilutions. MIC values were determined after 48 h of incubation for *Candida* spp. and 72 h for *Cryptococcus neoformans*. MLC were assessed immediately after MIC determination. Briefly, 20 μL from each well showing no visible growth were subcultured onto Sabouraud dextrose agar plates and incubated at 35 °C for an additional 48 h or 72 h, depending on the strain, to confirm the absence of regrowth. MIC and MLC values for *C. albicans* were also assessed in the presence of sorbitol, an osmoprotective agent, using a modified broth microdilution assay performed as reported above, in which sorbitol was incorporated into the culture medium to a final concentration of 0.8 M.

#### 4.3.3. *Candida albicans* Germ Tube Formation

To assess the effect of *F. silaifolia* and *F. cassia* essential oils on *C. albicans* germ tube formation, the yeast was initially grown for 24 h on Sabouraud dextrose agar plates. Cell suspensions of *C. albicans* ATCC 10231 were then prepared in NYP medium [N-acetylglucosamine (10^−3^ mol/L; Sigma-Aldrich, Darmstadt, Germany), Yeast Nitrogen Base (3.35 g/L; Fluka, Honeywell International, Seelze, Germany), proline (10^−3^ mol/L; Sigma-Aldrich, Darmstadt, Germany), NaCl [4.5 g/L; Merck, Merck KGaA, Darmstadt, Germany), pH 6.7 ± 0.1] and adjusted to a final concentration of 1 × 10^6^ CFU/mL. Volumes of 100 μL of the fungal suspension were added to each well of a 96-well microplate, followed by 100 μL of NYP medium containing the essential oils at various concentrations (ranging from MIC to MIC/64), yielding a final volume of 200 μL per well. Microplates were incubated at 37 °C for 3 h, after which images were captured for subsequent quantification of germ tube formation. For each condition, three independent experiments were performed, and a total of 150 fungal cells were counted per image. Germ tube-positive cells were defined as those exhibiting a germ tube with a length greater than the diameter of the mother cell. The percentage of germ tube formation was calculated based on the number of positive cells relative to the total count.

#### 4.3.4. *Candida albicans* Biofilm Formation and Disruption

The effect of the essential oils on *C. albicans* biofilm formation and disruption was assessed using a method previously described by Alves-Silva et al. [43]. In summary, 20 μL of *C. albicans* from a 24 h culture on SDA was transferred to YPD medium (1% yeast extract, 2% peptone, and 2% dextrose; Sigma-Aldrich, Darmstadt, Germany) and incubated at 37 °C for 24 h. The following day, the medium was removed, and the cells were washed twice with PBS and centrifuged at 3000× *g* for 10 min. A cell suspension (1 × 10^6^ CFU/mL) was then prepared in RPMI-1640 medium (Gibco, Thermo Fisher Scientific, Waltham, MA, USA), supplemented with MOPS (Sigma-Aldrich, Darmstadt, Germany) and glucose (Sigma-Aldrich, Darmstadt, Germany), according to EUCAST guidelines [44]). Subsequently, 200 μL of this suspension was added to sterile 96-well polystyrene microtiter plates and incubated at 37 °C for 3 h, to allow fungal adhesion. After adhesion, the medium was removed and the wells were washed with PBS.

For the biofilm formation assay, 200 μL of RPMI-1640 containing different concentrations of the essential oils (800–1.56 μg/mL), prepared from DMSO (Sigma-Aldrich, Darmstadt, Germany) stock solutions, were added to each well, followed by incubation at 37 °C for 72 h. For the biofilm disruption assay, following the adhesion phase, the medium was carefully removed and the wells were gently washed with PBS to eliminate non-adherent cells. Fresh RPMI-1640 medium (200 μL) was then added, and the plates were incubated at 37 °C for 72 h to allow biofilm maturation. Following this period, the medium was then removed, the wells were washed with PBS, and 200 μL of RPMI-1640 containing the essential oils the same concentration range (800–1.56 μg/mL) was added, followed by incubation for an additional 24 h. In both assays, appropriate controls were included: a positive control consisting of inoculated medium without essential oil (with DMSO at the highest concentration used in the test solutions) and a negative control consisting of medium only.

##### Biofilm Viability

Biofilm viability was assessed using the XTT metabolic assay, as previously reported [43]. Following incubation, the medium was removed and the wells were washed with PBS. Then, 100 µL of freshly prepared XTT (Sigma-Aldrich, Darmstadt, Germany) solution (1 mg/mL with 4 µM of menadione; PanReac AppliChem, Barcelona, Spain) was added to each well and the plate further incubated for 3 h at 37 °C. After this period, the absorbance at 450 nm was measured in an automated plate reader (SLT, Wolfurt, Austria). Metabolic activity was calculated using the following equation: Metabolic activity (%) = Abs treatment/Abs control × 100; being Abs treatment the absorbance of the treated fungal cells and Abs control the absorbance of oil-free fungal cells. Experiments were performed in triplicate with two replicates per condition.

##### Biofilm Biomass

In order to assess biofilm biomass, biofilms were stained using crystal violet (Sigma-Aldrich, Darmstadt, Germany) under identical handing and timing conditions [43]. Briefly, following medium removal, the wells were gently washed with PBS, and all residual liquid was completely removed. Then biofilms were fixed with methanol (Sigma-Aldrich, Darmstadt, Germany) for 10 min and 100 μL of 0.5% crystal violet solution was added and incubated for 15 min. The wells were washed twice with sterile water to remove excess stain. Next, 150 μL of 33% acetic acid (Sigma-Aldrich, Darmstadt, Germany) was added to solubilize the bound dye. The resulting solution was transferred to new wells, and the absorbance was measured at 620 nm. Biomass production was calculated using the following equation: Biomass (%) = AbsT/AbsC × 100; being AbsT the absorbance of the treated biofilms and *AbsC* the absorbance of oil-free biofilms. Experiments were performed in triplicate with two replicates per condition.

##### Extracellular Matrix

Extracellular matrix deposition was quantified using safranin red (Sigma-Aldrich, Darmstadt, Germany) staining [43]. Following medium removal, the biofilms were washed with PBS, and 100 μL of 0.5% safranin solution was added and incubated for 5 min. The biofilms were then washed twice with PBS to remove excess stain. Finally, 33% acetic acid (Sigma-Aldrich, Darmstadt, Germany) was added to solubilize the bound dye, and the resulting solution was transferred to new wells. Absorbance was measured at 520 nm. ECM deposition was calculated using the following equation: Extracellular matrix (%) = AbsT/AbsC × 100; being AbsT the absorbance of the treated biofilms and AbsC the absorbance of oil-free biofilms. Experiments were performed in triplicate with two replicates per condition.

### 4.4. Wound Healing Potential

#### 4.4.1. Cell Culture

Mouse fibroblasts (NIH/3T3 cell line, ATCC CRL-1658, Manassas, VA, USA) were cultured on Dulbecco’s Modified Eagle Medium (DMEM 12800-017; Gibco, Waltham, MA, USA), supplemented with 10% (*v*/*v*) heat-inactivated fetal bovine serum (FBS; Gibco, Waltham, MA, USA) and 1% (*v*/*v*) penicillin/streptomycin solution (Gibco, Waltham, MA, USA). Human keratinocytes (HaCaT cell line) were cultured on Dulbecco’s Modified Eagle Medium (DMEM 31600-083; Gibco, Waltham, MA, USA), supplemented with NaHCO_3_ (3.7 g/L, Sigma-Aldrich, Darmstadt, Germany), 1% (*v*/*v*) penicillin/streptomycin solution (Gibco, Waltham, MA, USA), 10% (*v*/*v*) heat-inactivated FBS (Gibco, Waltham, MA, USA) and glucose (3.7 g/L, Sigma-Aldrich, Darmstadt, Germany). Cells were maintained in a humidified 5% CO_2_ 95% air atmosphere at 37 °C and subcultured at 80% confluence. For fibroblasts, trypsin (Sigma-Aldrich, Darmstadt, Germany), was used to detach the cells, whereas keratinocytes were treated with PBS–ethylenediaminetetraacetic acid (EDTA) followed by Triple Express (Alfagene, Paço de Arcos, Portugal). Cell morphology was controlled using a VWR VisiScope IT417PH inverted optical microscope (VWR International, Galdenaaksebaan, Leuven, Belgium).

#### 4.4.2. Cell Viability

The safety profile of the essential oil was assessed using the resazurin reduction assay, as previously reported [45,46]. Briefly, 150 μL of a cell suspension (1.33 × 10^5^ cells/mL for fibroblast and 1.5 × 10^5^ cells/mL for keratinocytes) were seeded into 96-well plates and incubated at 37 °C. After an overnight stabilization period, cells were exposed to different concentrations of the essential oils (800–1.56 µg/mL) and further incubated for 24 h. Subsequently, the medium was removed, and the wells were washed with PBS. Then, 150 μL of resazurin (Sigma-Aldrich, Darmstadt, Germany) solution (500 µM; 1:10 dilution in DMEM, Gibco, Waltham, MA, USA) was added to each well and incubated for 2 h at 37 °C. Both untreated and DMSO (Sigma-Aldrich, Darmstadt, Germany) controls were included, together with blank wells containing resazurin without cells. Absorbance was measured at 570 nm and 620 nm using an automated plate reader (SLT, Austria). Cell viability was calculated using the following equation: [(AbsT 520 − AbsT 620) − (AbsR 520 − AbsR 620)]/[(AbsC 520 − AbsC 620) − (AbsR 520 − AbsR 620) × 100; where AbsT, AbsC, and AbsR correspond to treated cells, untreated (oil-free) control cells, and resazurin-only blanks (without cells), respectively. Background absorbance at 620 nm was subtracted from the corresponding 520 nm readings for all measurements. Experiments were performed in triplicate.

#### 4.4.3. Cell Migration

The scratch wound assay was performed as previously described [46]. Briefly, cells were seeded at a density of 3 × 10^5^ cells/mL in 12-well plates and incubated at 37 °C for 24–48 h until reaching approximately 80% confluence. A wound was created by scratching the cell monolayer with a 10 µL pipette tip (one scratch per well). Then, cells were washed with sterile PBS (pH 7.4), and different concentrations of the essential oil (100–25 µg/mL), prepared in DMEM (Gibco, Waltham, MA, USA) and supplemented with 2% FBS were added. Untreated control cells (without essential oil) were also included. Images of the wound area were captured at 0 h and 15 h post-scratch using a VWR VisiScope IT417PH inverted optical microscope (VWR International, Leuven, Belgium). The wound closure percentage was calculated for each condition relative to its own initial wound area (0 h). These values were then normalized to the untreated control, which was considered as 100% wound closure for comparison between groups [47]. Experiments were performed in triplicate.

### 4.5. Enzyme Activity

#### 4.5.1. Tyrosinase Activity

Tyrosinase activity was determined according to González-Vázquez et al. [46]. Kojic acid (10.6 mM; Sigma-Aldrich, St. Louis, MI, USA) was used as a reference standard. Briefly, 110 μL of PBS (0.1 M, pH 6.8), 10 μL of essential oil at different concentrations (800–12.5 μg/mL), 10 μL of mushroom tyrosinase (1500 U/mL; Sigma-Aldrich, Darmstadt, Germany), and 20 μL of L-tyrosine substrate (1.5 mM; Sigma-Aldrich, Darmstadt, Germany) were added to each well of a 96-well plate. The reaction mixture was incubated for 15 min at 37 °C and then placed on ice for 1 min to stop the reaction by rapid cooling. Absorbance was measured at 450 nm using a Multiskan FC microplate spectrophotometer (Thermo Fisher Scientific Inc., Waltham, MA, USA). Tyrosinase activity was calculated using the following equation: [(C − D)/(A − B) × 100, being C the absorbance of the sample with enzyme, D the absorbance of the sample without enzyme, A the absorbance of the positive control (with enzyme and without essential oil), and B the absorbance of the negative control (without enzyme and without essential oil). Experiments were performed in triplicate with three replicates per condition.

#### 4.5.2. Elastase Activity

Elastase activity was assessed according to the method reported by González-Vázquez et al. [46] with slight modifications. Quercetin (50 μg/mL, Sigma-Aldrich, Darmstadt, Germany) was used as a reference standard. Briefly, 100 μL of Tris–HCl buffer (0.1 M, pH 8.0, Sigma-Aldrich, Darmstadt, Germany), 25 μL of N-succinyl-Ala-Ala-Ala-p-nitroanilide (4.4 mM; Sigma-Aldrich, Darmstadt, Germany), and 50 μL of essential oil at different concentrations (800–12.5 μg/mL) were added to each well of a 96-well plate and incubated for 15 min at 25 °C. The absorbance was then measured at 410 nm. Subsequently, 25 μL of elastase enzyme solution (0.3 U/mL; Sigma-Aldrich, Darmstadt, Germany) was added, and the plate was incubated for an additional 15 min at 25 °C. Absorbance was measured again at 410 nm. Elastase activity was calculated using the following equation: [(C − D)/(A − B)] × 100, being C and D the absorbance of the essential oil-treated wells after and before enzyme incubation, respectively, and A and B, the absorbance of the untreated wells after and before enzyme incubation, respectively. Experiments were performed in triplicate with three replicates per condition.

## Figures and Tables

**Table 1:** Antifungal activity of *Ferulago silaifolia* and *Ferulago cassia* essential oils.

Strains	*Ferulago silaifolia* EO				*Ferulago cassia* EO			
	MIC	MLC	MIC50	MIC90	MIC	MLC	MIC50	MIC90
*Candida albicans* ATCC 10231	400	400	227.96	353.94	800	>800	597.31	773.52
*Candida tropicalis* ATCC 13803	800	800	400.03	773.16	>800	>800	>800	>800
*Candida krusei* H9	100	100	68.47	769.52	200	200	120.92	409
*Candida guilliermondii* MAT 23	100	100	65.04	711.59	200	200	110.15	328.56
*Candida parapsilosis* ATCC 90018	100	100	72.29	443.13	400	400	130.11	486.66
*Cryptococcus neoformans* CECT 1078	50	50	29.82	444.56	100	100	36.75	142.79

MIC and MLC values in μg/mL; EO—essential oil.

**Table 2:** Plant collection sites.

Species	Location
*F. cassia*	Konya: Beyşehir, Tınaztepe-Bozkır old road, 1st km, serpentine areas, in gladelike areas under *Pinus nigra* J.F.Arnold, 1555 m, 20 June 2016 (AEF 28775)
*F. silaifolia*	Bursa, Uludağ road, 100 km to National Park, under *Castanea sativa* Mill. and *Pinus nigra* forest, 837 m, 7 June 2016 (AEF 28771)
*F. isaurica*	Antalya-Alanya, between Durbannaz-Banlıca, under *Pinus brutia* forest, calcareous rocks, 837 m, 21 June 2016 (AEF 28778)
*F. setifolia*	Erzincan: Üzümlü district, Karakaya Town, between Karakaya-Tekçam Highland, 2100 m, high mountain steppe, 2047 m, 14 July 2016 (AEF 28770)
*F. syriaca*	Hatay: Harbiye-Şenköy road, ca. 5th–6th km, around maquis, 452 m, 22 June 2016 (AEF 28779)

## Data Availability

The data supporting the conclusions of this article will be made available by the authors on request.
